# Does the coagulation profile really matter in central venous cannulation? A review of the literature

**DOI:** 10.1186/cc9854

**Published:** 2011-03-11

**Authors:** J Baombe, L Sultan, B Foex

**Affiliations:** 1 Manchester Royal Infirmary, Manchester, UK

## Introduction

There is great variation in practice and opinions regarding the safety in inserting central venous lines in patients with coagulopathy. The authors reviewed the medical literature reporting the incidence of complications (haemorrhagic and nonhaemorrhagic) following the insertion under ultrasound guidance of a central venous line.

## Methods

The authors searched the MEDLINE and Embase databases for relevant terms. The MEDLINE database (1950 to week 2 December 2010) was explored with the terms central line, catheterization, coagulopathy, blood coagulation disorder, international normalized ratio, thrombocytopenia with their appropriate combinations and truncated terms. The Embase database (1980 to week 2 December 2010) was searched with the terms central venous catheter, blood clotting disorder, thrombocytopenia, international normalized ratio, complications with their appropriate combinations and truncated terms. Both searches were limited to English language, humans and adults only.

## Results

We found 413 papers with the MEDLINE search strategy. After abstract review and critical appraisal, only five articles were deemed to be directly relevant to our question and of level of evidence high enough to be considered. These were included in our final summary table. The Embase search returned 257 papers, only one relevant but also a duplicate from the previous search.

## Conclusions

The retrieved studies seem to suggest that the insertion of central lines under ultrasound guidance do not require correction of haemostatic abnormalities prior to intervention. Rates of haemorrhage are low in patients with elevated prothrombin time, activated partial thromboplastin time, international normalized ratio or low thrombocyte count and appear to be closely related to the level of experience of the physician rather than the defects of haemostasis.

